# A Narrative Review of Advancements in Understanding and Treating Varicose Veins

**DOI:** 10.7759/cureus.48093

**Published:** 2023-11-01

**Authors:** Aditi Singh, Rajesh Gattani

**Affiliations:** 1 Medical School, Jawaharlal Nehru Medical College, Datta Meghe Institute of Higher Education and Research, Wardha, IND; 2 General Surgery, Jawaharlal Nehru Medical College, Datta Meghe Institute of Higher Education and Research, Wardha, IND

**Keywords:** radiofrequency ablation, varicose veins, phlebectomy, endogenous laser ablation, vein ligation, stripping, sclerotherapy

## Abstract

Chronic venous disease, with varicose veins as its archetypal manifestation, stands as a pervasive and intricate health quandary, encompassing a vast array of contributing factors. Age, genetics, obesity, pregnancy, and prolonged immobility weave a complex tapestry, underscoring the omnipresence of this ailment. Its societal and economic footprint is undeniably formidable, as diverse classifications underscore its multifaceted character. The intricate interplay of chronic venous disease with diabetes mellitus and neuropathy compounds the challenge, fostering soaring healthcare expenditures and a palpable erosion of quality of life, particularly among women harboring cardiometabolic risk factors. Despite research shedding light on heightened susceptibility within certain demographics, the enigmatic determinants orchestrating the transition from mild to severe chronic venous disease continue to elude us.

Varicose veins, marked by the presence of dilated and tortuous subcutaneous vessels, precipitate both physical discomfort and cosmetic concerns, frequently necessitating meticulous clinical evaluation coupled with ultrasound studies to secure a precise diagnosis. Treatment strategies are strategically crafted to ameliorate distressing symptoms, enhance aesthetic concerns, and forestall potential complications. Nevertheless, the prognostication of chronic venous disease remains ensconced in a degree of ambiguity, hinting at the vast terrain yet to be charted in this medical domain. The quest to fathom the intricacies of this condition uncovers an ever-evolving panorama where conservative interventions play an indispensable role in managing mild cases, while interventional procedures like endovenous laser ablation and sclerotherapy step onto the stage for patients grappling with severe symptoms, thus treading the fine line between efficacy and invasiveness. Moreover, a meticulous economic analysis underscores the cost-effectiveness of various therapeutic modalities, thereby bolstering the imperative of a patient-centered approach. As we navigate the labyrinthine complexities of chronic venous disease and varicose vein management, we are inexorably drawn to the pivotal role of customized treatment approaches, as well as the dynamic interplay between scientific progress, patient preferences, and therapeutic innovations in the relentless pursuit of optimized outcomes and an enhanced quality of life.

## Introduction and background

Chronic venous disease looms as an alarmingly pervasive and intricate health concern, casting its shadow over millions of individuals and, alas, often revealing itself only in its advanced stages, complicating the path to effective intervention. At the heart of this condition lies the omnipresent manifestation of varicose veins, a visual marker of venous insufficiency that spans a wide-ranging spectrum, with their prevalence oscillating between 5% and 30% in the general population. Traditionally, a striking female predilection was observed, with a reported female-to-male ratio of 3:1 [1]. However, recent data tantalizingly hint at the possibility of a more equitable gender distribution in the prevalence of this vascular disorder, underlining the ever-evolving nature of our understanding.

The intricate web of risk factors contributing to the emergence of this multifaceted ailment includes the inevitable passage of time, the profound influence of genetic predisposition, the burdensome weight of obesity, the miraculous yet taxing journey of pregnancy, the specter of past phlebitis episodes, the haunting legacy of leg injuries, and the relentless demands of prolonged periods of standing or sitting in the workplace [2]. Chronic venous disease's repercussions are substantial, with up to 50% of affected individuals exhibiting various venous abnormalities that go beyond varicose veins alone. This diverse landscape of prevalence serves as a stark reminder of the considerable societal and economic impact of chronic venous disease. When considering the multidimensional framework of clinical, etiological, anatomical, and pathophysiological classifications, we invariably unearth a more profound socioeconomic burden that permeates the fabric of healthcare [2,3].

To further compound the challenge, the convergence of chronic venous disease with other medical conditions, such as diabetes mellitus, ushers in a more formidable adversary. The presence of diabetic neuropathy accentuates the complexity of management, resulting in augmented healthcare costs and a perceptible erosion in the quality of life for those grappling with this dual burden. Recent investigations have shed light on the heightened vulnerability of females bearing cardiometabolic risk factors or diabetes to the early onset of chronic venous disorders. Nevertheless, the intricate determinants that guide the transition from mild to severe forms of chronic venous disease remain primarily cloaked in mystery, a veil that researchers and clinicians ardently seek to lift [3].

Varicose veins, the hallmark of chronic venous disease, are present as dilated, tortuous subcutaneous veins, most commonly adorning the lower limbs, their vibrant colors often resembling nature's palette of green, dark blue, or regal purple. Their emergence can be attributed to the insufficiency of venous valves and the enfeeblement of vein walls, a tandem malfunction that conspires to facilitate pooling and retrograde blood flow, exacerbating the condition [4]. Achieving an accurate diagnosis hinges on the judicious deployment of clinical examination techniques combined with Doppler or duplex ultrasound studies to scrutinize for evidence of venous reflux.

The clinical tableau painted by varicose veins is one of discomfort, encompassing an array of symptoms that add to the burden of this condition. Patients may endure pain, pruritus, a persistent sense of ponderousness in their limbs, nocturnal cramps that disrupt their rest, and, not insignificantly, the anxiety brought on by the cosmetic impact of these conspicuous veins [5]. Yet, just like the broader chronic venous disease, the incidence and prevalence of varicose veins exhibit marked heterogeneity across diverse populations. Potential risk factors implicated in their onset encompass sustained periods of upright posture and the imposition of abdominal masses, as encountered in pregnancy or engendered by the burden of obesity. While specific studies have illuminated certain risk factors, including the crucibles of multiple pregnancies, obesity, and protracted standing or ambulation in the occupational realm, the tapestry of risk factors remains incomplete, with lacunae yet to be filled by a more exhaustive understanding of this multifaceted condition [5,6].

Numerous interventions have been crafted to assuage the symptoms, restore aesthetic harmony, and mitigate the specter of complications that loom over varicose veins and chronic venous disease alike. These interventions are measured against a spectrum of parameters, including the alleviation of symptoms, the enhancement of the quality of life, the recurrence rate, and the constellation of treatment-related complications, such as the specter of hematoma formation or the ominous shadow of thromboembolic events [7]. Yet, despite these interventions, the prognosis remains a nebulous terrain with a conspicuous lack of clarity. The frequency of complications remains a subject that beckons for further illumination as it perpetuates the quest for enhanced patient care and outcomes [7].

As we navigate this intricate landscape of chronic venous disease and its varicose vein manifestations, we confront not just the challenges of diagnosis and treatment but also the realization that this complex canvas, marked by its multifaceted tapestry of risk factors and outcomes, still harbors many secrets. These mysteries await the unmasking light of future research to unravel the intricacies and unveil the concealed truths that continue to enshroud these conditions. The journey is a testament to the unrelenting pursuit of knowledge and the unwavering commitment to enhancing the quality of life for those affected by these conditions, even as we acknowledge the depths of our current understanding.

## Review

Methodology

Search Strategies

We performed a comprehensive search in the electronic databases PubMed, MEDLINE, Excerpta Medica Database (Embase), Google Scholar, and ResearchGate and an examination of the English-language literature.

Inclusion Criteria

We included peer-reviewed journals produced in English, articles published in the last 15 years, the full text of the publication, publication categories like review articles, systematic reviews, meta-analyses, or empirical studies published in peer-reviewed scientific journals, and in compliance with the combinations of keywords such as "varicose veins", "radiofrequency ablation", "phlebectomy", "endogenous laser ablation", "vein ligation", "stripping", and "sclerotherapy".

Exclusion Criteria

We excluded articles published earlier than 15 years, lack of full text of the publication, language of the publication different than English, type of publication different than review articles, systematic review, meta-analysis, or empirical studies published in peer-reviewed scientific journals, malignancy, co-morbidities along with the diseases affecting presentation, specific treatment protocol, or that of the illness in review.

**Figure 1:**
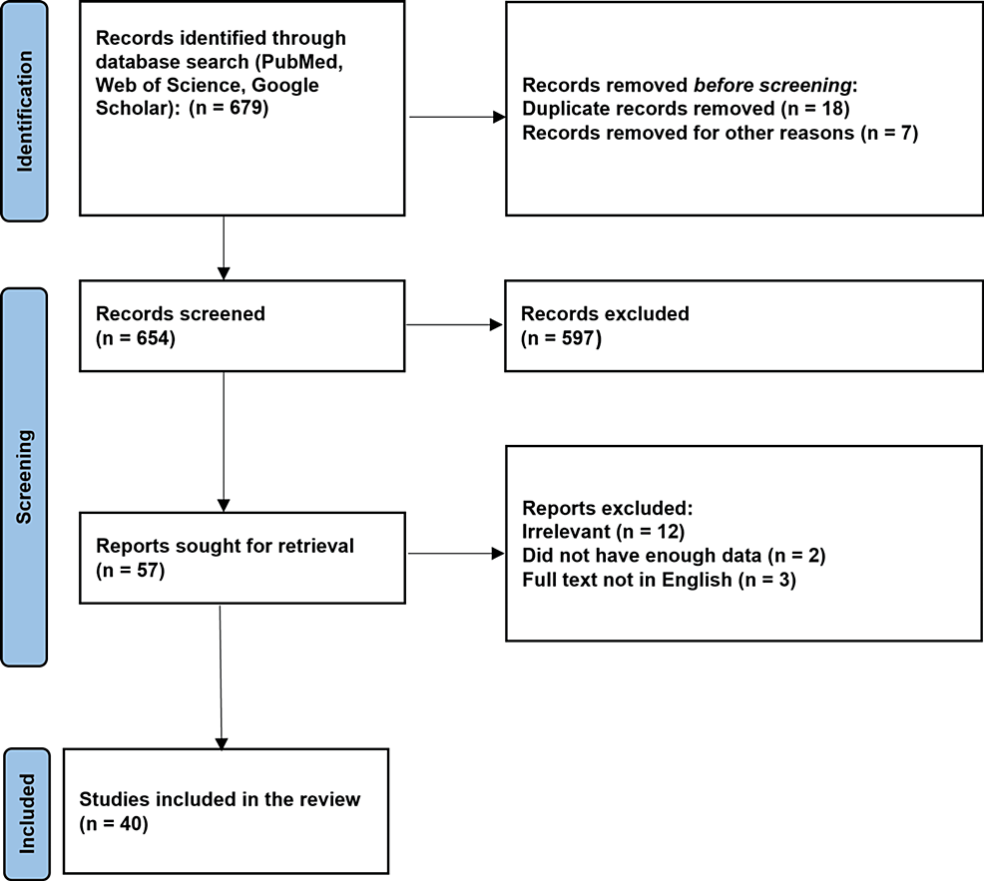
The selection process of articles used in this study Preferred Reporting Items for Systematic Review and Meta-Analysis (PRISMA) flowchart for the keywords used in the literature review.

Key terms used for the search are "varicose veins" (All Fields) OR "varicose"(All Fields) AND "radiofrequency ablation" (All Fields) OR "varicose veins" (MeSH Terms) OR "treatment of varicose veins"[All Fields]. The Preferred Reporting Items for Systematic Reviews and Meta-Analyses (PRISMA) guidelines used in research methodology are depicted in Figure 1.

Patient approach

When patients embark on the path to seek treatment for varicose veins, the importance of a meticulous and comprehensive assessment cannot be overstated. While a clinical examination can offer valuable insights, it is often the judicious use of Doppler ultrasound that serves as the cornerstone for a thorough evaluation. This widely employed diagnostic technique allows healthcare professionals to delve deep into the intricacies of venous dynamics by scrutinizing blood flow during specific maneuvers. Of particular significance is the identification of sites prone to valve incompetence, with the saphenofemoral junction in the groin being a focal point where such incompetence is more frequently encountered. The integration of Doppler ultrasound into the diagnostic process ensures a precise understanding of the patient's condition, paving the way for informed decisions regarding the most suitable treatment options [6].

The choice of treatment modalities hinges on the nature of the varicose veins and the underlying physiological intricacies. In instances where valve incompetence is not detected in upstream regions and the issue predominantly resides below the knee, injection treatment in the form of compression sclerotherapy emerges as a compelling option. This non-invasive approach yields promising results, significantly alleviating symptoms and cosmetic concerns. However, it is important to acknowledge that due to the underlying valve issues, the specter of recurrence looms over these cases. For those confronted with more complex varicose vein scenarios, characterized by the incompetence of valves between deep and superficial veins, surgery offers a pathway to enduring relief. Surgical interventions typically involve the precise division of superficial veins at the sites of incompetence, the stripping of long saphenous veins, and the meticulous removal of varicosities through discreet small incisions. It is worth noting that bilateral varicose vein operations can be a time-consuming endeavor, necessitating a collaborative team approach to ensure the best possible outcomes. Additionally, the prioritization of patients with complications such as skin changes or ulcers is paramount, as their conditions warrant specialized attention and timely interventions [6,7].

Simultaneously, the realm of varicose vein treatment encompasses individuals with different needs and aspirations. Patients grappling with concerns of a cosmetic nature or mild discomfort deserve the same level of expert assessment to tailor their treatment strategies effectively. Their journey involves a nuanced evaluation that takes into account local circumstances and individual preferences. The reassuring guidance provided by healthcare professionals plays a pivotal role in this phase, ensuring that patients are well-informed about their options and potential outcomes. For those individuals presenting with harmless varicose veins and expressing the desire for treatment, reassurance is coupled with a range of therapeutic alternatives. This includes the prospect of temporary symptom alleviation through sclerotherapy or the option to await surgical intervention if they are more inclined towards a lasting solution. In cases where a patient's eagerness for active treatment persists even after being assured of the benign nature of their condition, a prudent referral to a hospital-based setting is a recommended course of action. This comprehensive approach to patient care is underpinned by a commitment to providing tailored solutions and compassionate support throughout the varicose vein treatment journey [7].

Assessment and decision-making

In the realm of assessing and determining the most suitable course of action for individuals dealing with varicose veins, venous duplex ultrasound has emerged as the gold standard. This advanced diagnostic tool has revolutionized the investigation of varicose veins, enabling a comprehensive evaluation of both deep and superficial systems, including critical junctions and perforators. Its role is pivotal in deciphering the patency and competence of the venous network. With a nuanced understanding of the underlying pathophysiology, healthcare professionals are well-equipped to make informed decisions tailored to the specific needs and complications of each patient [6,7].

However, the approach to varicose vein management is not one-size-fits-all, and it must be fine-tuned to the individual's unique circumstances. When faced with uncomplicated, painless veins, providing reassurance often suffices. It's noteworthy that in the age of readily accessible information and the influence of anecdotal advice, some patients may be inclined to pursue specific treatments driven by recommendations from acquaintances or online research, even in the absence of clear medical indications. In these instances, the expertise of specialists becomes invaluable, ensuring that decisions are rooted in sound medical judgment rather than anecdotal evidence [2,4,7].

Within the domain of public health systems, there is an ongoing need for discourse regarding the management of uncomplicated varicose veins. Guidelines advocate for conservative approaches, reserving more invasive treatments for advanced cases or when debilitating symptoms are present. The key lies in striking a balance between the principles of conservative management and the necessity for intervention, guided by the unique needs of each patient. Patients grappling with varicose vein complications such as venous eczema, ulcers, insufficiency, thrombophlebitis, bleeding, or discomfort should find their path leading to a vascular team. This is a critical step in ensuring that specialized care is administered, addressing the complexities of their conditions effectively [8].

Pre-referral discussions between healthcare professionals and patients are valuable, particularly in scenarios where individuals might harbor reservations about further interventions. These dialogues help in allaying concerns, elucidating the potential benefits of treatment, and addressing any lingering doubts. For those experiencing symptoms and whose foot pulses remain palpable or possess an ankle-brachial index abnormality above 0.6, below-the-knee compression stockings, often of class 2, can offer substantial relief and symptom management. It is a conservative yet effective approach to enhancing the patient's quality of life. The landscape of treatment options is further shaped by patient comorbidities, influencing the suitability of various interventions. Typically, surgical treatments are reserved for those who, apart from their varicose veins, grapple with severe medical conditions or obesity. Exceptions to this rule exist in cases where non-healing venous ulcers are a prevailing concern, warranting a more aggressive approach to care [7,8].

**Figure 2:**
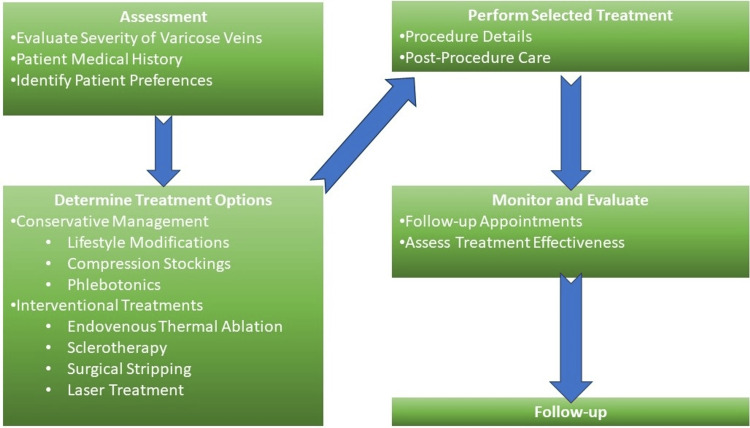
Management strategy in the treatment of varicose veins Figure credits: Aditi Singh (author)

In the intricate realm of varicose vein management, the assessment and decision-making process is as diverse as the patients themselves, necessitating a nuanced approach that blends state-of-the-art diagnostics with a patient-centric ethos. It's through these carefully considered decisions that individuals receive the personalized care they require, ensuring that their varicose vein journey is one of empowerment and effective intervention. Future developments in the endovenous field might influence this approach [9]. The general management strategy for the patient is given in Figure 2.

Conservative management: a pillar of varicose vein care

External compression therapy remains a cornerstone in the holistic approach to managing varicose veins. This therapeutic approach involves various tools, including bandages, support/compression stockings, and intermittent compression devices. These methods have consistently demonstrated their efficacy in alleviating the complications associated with this condition and can be employed either as standalone treatments or in conjunction with other interventions [10]. However, it is imperative to acknowledge that compression therapy may not be suitable for every case. Contraindications include individuals with insufficient vascular functionality, active skin diseases, or allergies to any materials incorporated in compression stockings. Despite these considerations, it remains a cost-effective treatment option with favorable results [11].

Overcoming Challenges: Customization and Compliance

Challenges may arise with compression therapy, primarily related to patient compliance. Some individuals find compression stockings cumbersome, time-consuming, and uncomfortable. Poor compliance can compromise treatment outcomes. Therefore, healthcare professionals should consider these challenges and provide guidance and support to enhance patient adherence [12]. Compression stockings are available in various brands, each offering different compression levels, typically 20-50 mmHg. Patients with varicose veins often require compression of 30-40 mmHg. Over-the-counter compression stockings usually offer comparatively lower compression levels, potentially insufficient for patients with more severe cases. Hence, precise measurement and fitting are paramount, and custom-fitted stockings may sometimes be necessary. It is also crucial to educate patients on the periodic replacement of compression garments due to the natural loss of elasticity over time [13].

Drug Therapy: A Limited but Promising Avenue

While drug therapy for varicose veins has limitations, there are promising options that deserve consideration. Traditional diuretics, used for managing edema, fall short of providing substantial relief from the pain and discomfort associated with varicose veins. However, horse chestnut extract, derived from the seeds of the horse chestnut tree (*Aesculus hippocastanum*), has emerged as a potential solution [14]. This natural remedy has shown promise for reducing edema by enhancing venous tone and flow. It has a long history of use in Europe for varicose vein treatment. A randomized controlled study comparing drug therapy with compression therapy revealed encouraging results. Patients treated with either compression stockings or horse chestnut extract experienced a significant improvement in their condition compared to a placebo after a few weeks of therapy. Moreover, this drug therapy, utilizing horse chestnut extract, was generally well-tolerated, with minimal reported adverse effects, although some cases of gastrointestinal discomfort were noted [15].

Laser and Pulsed-Light Therapy: Advancements and Efficacy

Laser and pulsed-light therapies have been employed to address spider veins in the legs for an extended period. Initial outcomes were occasionally dissatisfying, mainly due to side effects such as pain, scarring, and recurrence. However, advancements in laser technology over the past 15-20 years have significantly improved the safety and efficacy of these treatments [16,17]. These therapies are particularly suitable for treating isolated small spider veins that are not associated with varicosities. They can also serve as complementary treatments following procedures such as endovenous chemical ablation or surgical interventions [18]. Patients who are averse to needles, have allergies to sclerotherapy, experience undesirable sclerotherapy effects, have matted telangiectasias, or possess delicate superficial spider veins below the malleolus level may be ideal candidates for these procedures [18,19]. Before opting for laser therapy, addressing any underlying venous reflux issues is crucial. Multiple sessions are typically required, with intervals ranging from six to 12 weeks. To optimize the treatment's effectiveness, patients should avoid sun exposure before the procedure due to potential risks associated with melanin absorption [19].

While both laser and pulsed-light therapies are generally well-tolerated, they may entail side effects, including pain, edema, erythema, bruising, pigmentation changes, and, rarely, scarring. The advent of improved lasers with longer wavelengths has substantially enhanced results [20]. Vascular lasers heat hemoglobin within the vessels, leading to endothelial damage and sclerosis. It is important to note that laser and light therapy can be relatively more expensive than sclerotherapy due to equipment costs. Different types of lasers are available, each varying in effectiveness, necessitating that physicians be proficient in their application [21]. In contrast, pulsed-light therapy, which emits a spectrum of light rather than a single wavelength, was introduced in the 1990s. It is effective for venous telangiectasias, although outcomes may be less predictable than laser treatment or endovenous chemical ablation [22, 23].

Evolving approaches to surgical management of varicose veins

Varicose veins, characterized by swollen and twisted veins often seen in the legs, are a common vascular condition that can lead to discomfort and cosmetic concerns. In this in-depth exploration, we examine the array of surgical treatments available for varicose veins, examining their effectiveness, advantages, and considerations. We also scrutinize the evolving landscape of surgical techniques and technologies, shedding light on the complexities and advancements in the field [24].

Saphenous Vein Ligation: The Gold Standard

Saphenous vein ligation, considered the gold standard in surgical treatment, involves the removal of the long or short saphenous veins and ligating (tying off) the junctions where these veins meet the femoral and popliteal veins. It is a well-established procedure indicated for patients with incompetent long or short saphenous veins, reflux through saphenofemoral or saphenopopliteal junctions, or superficial thrombophlebitis, identified through duplex Doppler ultrasound [25]. Traditionally, there was a belief that stripping the saphenous vein from the ankle to the groin was necessary to address the reflux issue that contributes to varicose vein formation. However, modern insights have evolved this notion, suggesting that ligating the saphenous vein at the saphenofemoral junction and removing the femoral portion of the vein can effectively resolve these issues. This approach can be combined advantageously with other treatments, such as endovenous chemical ablation [26].

Endovenous chemical ablation, often employed following endovenous laser ablation, aims to manage any remaining abnormal dilatations. The procedure is well-suited for effectively addressing more prominent varicose veins, particularly in the trunk region, where high blood flow may limit the effectiveness of alternative treatments like sclerotherapy [27]. Ambulatory or stab phlebectomy is a straightforward, cost-effective method for removing dilated and tortuous veins. This procedure is particularly suitable for removing more prominent varicose veins, a notable advantage in managing such cases [28]. Ambulatory phlebectomy has shown effectiveness, especially for more prominent abnormally dilated veins in regions with robust blood flow, where other treatments might be less productive. It also benefits patients with thicker vein walls, such as younger individuals [28, 29]. The procedure is often performed with other surgical methods, such as saphenous ligation at the saphenofemoral and saphenopopliteal junctions. The varicosity is surgically clamped during ambulatory phlebectomy and extracted in smaller segments. While complications are generally rare, they may include bleeding, hematomas (blood collections outside blood vessels), transient hyperpigmentation, infection, pain, and occasional telangiectatic matting, which refers to the appearance of new small blood vessels [29].

Endovenous Saphenous Vein Obliteration: A Minimally Invasive Option

Endovenous saphenous vein obliteration represents an advanced surgical technique to reduce the invasiveness of traditional varicose vein treatments. The procedure utilizes endovenous thermal ablation, employing lasers or high-frequency radio waves to generate higher temperatures. Laser ablation transfers heat to the vessel wall [30]. In contrast, radio-frequency ablation directs heat directly to the vein wall, potentially reducing the risk of skin staining due to residual blood products. Many practitioners consider the combination of endovenous thermal ablation with saphenofemoral junction ligation beneficial [31]. However, ongoing debates surround the necessity of this additional surgical step. Tumescent anesthesia is crucial for this procedure, injecting a mixture of local anesthetics, such as lidocaine and normal saline, along the dilated vein. This anesthesia facilitates the performance of the system in an office setting. Notably, endovenous obliteration effectively closes off the vein while preserving its integrity, reducing the risk of hematoma formation, and minimizing skin discoloration and pain [31,32].

Duplex Doppler ultrasonography is indispensable before and during surgery to ensure precise visualization and planning. Specific vein diameter and depth requirements must be met for the procedure. The depth requirement, in particular, limits the procedure's applicability in the calf and ankle regions. Access to the greater saphenous vein is typically established just below the knee crease through a venous cut-down or direct puncture using a large-bore needle. Recent developments have expanded the range of options by enabling punching at the ankle, providing additional treatment possibilities [33].

Transilluminated Power Phlebectomy: Minimizing Discomfort and Scarring

Transilluminated power phlebectomy is tailored to treat varicose veins in the thigh region. Distinguishing itself from stab phlebectomy, which entails multiple incisions and potential pain, hematomas, and scarring, transilluminated power phlebectomy aims to reduce complications by minimizing the number of incisions while effectively removing varicose veins [33]. During this procedure, a fiberoptic light channel equipped with a side port is inserted to provide illumination while infusing saline and local anesthetic. This creates tumescence (swelling) in the subcutaneous space, allowing vein visualization through transillumination [34]. The procedure involves a powered tissue resection, guided by the illuminated silhouette of the veins, to fragment and suction out the varicose veins [34]. Tumescent anesthesia controls bleeding and postoperative pain, while small incisions reduce scarring. Early data from studies suggest that this technique yields improved pain scores, cosmetic results, and patient satisfaction compared to traditional stab phlebectomy. Complications that arise post-surgery, such as cellulitis and hematomas, are generally self-limiting and do not require extensive treatment [35].

The surgical management of varicose veins has seen significant developments over the years, offering patients a range of options tailored to their specific needs. Traditional procedures like saphenous vein ligation and ambulatory phlebectomy continue to be effective treatments for varicose veins, while newer techniques like endovenous saphenous vein obliteration and transilluminated power phlebectomy are enhancing the field with their minimally invasive and patient-friendly approaches [36,37]. When selecting the most appropriate surgical intervention, healthcare providers and patients must consider individual circumstances and preferences. As vascular surgery continues to evolve, patients with varicose veins can look forward to more refined and less invasive treatments, ultimately improving their quality of life [37,38].

Overall analysis of the available treatment options

The excerpt provided by some researchers across the United States of America and the United Kingdom discusses a detailed economic analysis and comparison of different treatment options for varicose veins. The study evaluates the costs and effectiveness of various interventions over a specific period for varicose veins [36,37]. Comparison of Interventions suggests that several studies compare different interventional procedures for treating varicose veins, including ultrasound-guided foam sclerotherapy, radiofrequency ablation, conventional surgery, mechanochemical ablation, endovenous laser ablation, high ligation and stripping, and cyanoacrylate closure. Results of the research indicate that the interventional procedure, ultrasound-guided foam sclerotherapy, is the least expensive but also the least effective, leading to a higher probability of intervention and treatment. Radio-frequency ablation is compared to conventional surgery, and the cost is calculated, indicating that radio-frequency ablation is considered a cost-effective method [38]. Consideration of multiple options gives information regarding analysis and acknowledges that mechano-chemical ablation, endovenous laser ablation, and high ligation and stripping are mentioned to have similar costs and outcomes, which might make them comparable in value for money. The analysis aims to provide insights into the economic considerations of different treatment options for varicose veins, helping clinicians and policymakers make informed decisions based on cost-effectiveness and patient outcomes [39,40]. Table 1 gives a comparison between different treatment options for varicose veins.

**Table 1:** Comparison between different treatment options for varicose veins This table gives a comparison of various treatment modalities available for varicose veins [23, 25, 27, 33, 34, 36].

Treatment Option	Description	Suitability	Pros	Cons
Conservative management	Lifestyle modifications, compression stockings, and phlebotonics	Mild cases, pregnant patients, those unwilling to undergo surgery	Non-invasive, minimal risk	May not provide long-term relief
Endovenous laser ablation	Uses heat to close the affected veins	Suitable for most cases, especially with severe symptoms	Highly effective, minimal scarring	Potential for pain during recovery
Sclerotherapy	Injection of a solution to collapse and close veins	Smaller veins, cosmetic concerns	Effective for smaller veins, minimal downtime	May require multiple sessions
Surgical stripping	Removal of the affected vein through surgery	Severe cases, large veins	Immediate relief, but invasive	Longer recovery, scarring
Laser ablation treatment	Uses laser energy to seal veins	Smaller to medium-sized veins	Less invasive, minimal scarring	Potential for post-treatment pigmentation

Results and discussion

Conservative Treatment

Conservative management options include lifestyle modifications, compression stockings, and phlebotonics. These methods suit mild cases, pregnant patients, and individuals unwilling to undergo surgery. They offer a non-invasive approach with minimal risk. However, they may not provide long-term relief, making them more appropriate for managing mild symptoms and preventing further deterioration [22,23].

Endovenous Laser Ablation

Endovenous laser ablation is a highly effective treatment option for patients with severe varicose vein symptoms. It involves using heat to close the affected veins, resulting in minimal scarring. While this method offers significant benefits, some patients may experience post-treatment pain during recovery [25,26].

Sclerotherapy

Sclerotherapy is the injection of a solution to collapse and close smaller veins, making it a viable option for patients with smaller veins or cosmetic concerns. It is effective for smaller veins, and downtime is minimal. However, multiple sessions may be necessary for optimal results [28,30].

Surgical Stripping

Surgical stripping is recommended for severe cases with prominent veins. It provides immediate relief but is an invasive procedure with longer recovery times and scarring [28,30].

Laser Ablation Treatment

Laser ablation treatment is more suitable for minor to medium-sized veins and is less invasive, resulting in minimal scarring. However, patients should know the potential for post-treatment pigmentation [33,38,39].

Economic Analysis

An economic analysis compared the costs and effectiveness of different varicose vein treatment options over a specified period. Various interventional procedures were assessed, including ultrasound-guided foam sclerotherapy, radiofrequency ablation, conventional surgery, mechanochemical ablation, endovenous laser ablation, high ligation and stripping, and cyanoacrylate closure [29,40].

The study found that ultrasound-guided foam sclerotherapy is the least expensive and most effective treatment option, leading to a higher probability of additional interventions. In contrast, radiofrequency ablation was considered cost-effective. Mechanochemical ablation, endovenous laser ablation, and high ligation and stripping were found to have similar costs and outcomes, making them comparable in terms of value for money. Varicose vein management requires a tailored approach, considering the patient's condition, preferences, and the available treatment options. Conservative management is a non-invasive starting point, particularly for mild cases, pregnant patients, and those opposed to surgery. For patients with severe symptoms and more prominent veins, interventional procedures like endovenous laser ablation, sclerotherapy, surgical stripping, and laser ablation treatment offer effective solutions [2,38,40].

## Conclusions

In this narrative exploration, we have meticulously traversed the multifaceted realm of chronic venous insufficiency and the therapeutic avenues for varicose veins. Our journey commenced by shedding light on the prevalence of chronic venous disease, a condition most prominently manifesting as varicose veins and elucidating the intricate web of risk factors intricately woven into the fabric of these vascular disorders. Our scholarly voyage then ventured into the diverse spectrum of treatment modalities, encompassing established surgical procedures like saphenous vein ligation and ambulatory phlebectomy while embracing the evolution of medical science through contemporary techniques such as endovenous saphenous vein obliteration and transilluminated power phlebectomy. Moreover, we navigated the financial landscape of varicose vein treatments, dissecting the pivotal realm of cost-effectiveness and therapeutic outcomes. Throughout this odyssey, one constant remained evident: the paramount significance of precise diagnosis, comprehensive patient assessment, and tailoring treatment plans to meet everyone’s unique needs, preferences, and medical necessities. It is unequivocally clear that the management of chronic venous disease is an endeavor of profound complexity, demanding an integrated approach that harmonizes the patient's journey with the relentless march of medical progress, continuously reshaping the landscape of varicose vein treatment and, in doing so, bestowing upon patients a broader array of options and elevated prospects for enhanced outcomes.

## References

[1] Eberhardt RT, Raffetto JD (2005). Chronic venous insufficiency. Circulation.

[2] Tisi PV (2011). Varicose veins. BMJ Clin Evid.

[3] Campbell WB (1990). Varicose veins. BMJ.

[4] Davies AH (2019). The seriousness of chronic venous disease: a review of real-world evidence. Adv Ther.

[5] Hobbs JT (1991). ABC of vascular diseases. Varicose veins. BMJ.

[6] Youn YJ, Lee J (2019). Chronic venous insufficiency and varicose veins of the lower extremities. Korean J Intern Med.

[7] Gloviczki P, Comerota AJ, Dalsing MC (2011). The care of patients with varicose veins and associated chronic venous diseases: clinical practice guidelines of the Society for Vascular Surgery and the American Venous Forum. J Vasc Surg.

[8] Tremblay J, Lewis EW, Allen PT (1985). Selecting a treatment for primary varicose veins. Can Med Assoc J.

[9] Delis KT, Husmann M, Kalodiki E, Wolfe JH, Nicolaides AN (2001). In situ hemodynamics of perforating veins in chronic venous insufficiency. J Vasc Surg.

[10] Gao RD, Qian SY, Wang HH, Liu YS, Ren SY (2022). Strategies and challenges in treatment of varicose veins and venous insufficiency. World J Clin Cases.

[11] Raetz J, Wilson M, Collins K (2019). Varicose veins: diagnosis and treatment. Am Fam Physician.

[12] Tickle J, Ovens L, Mahoney K, Hunt S, Harris E, Hodgman L (2017). A proven alternative to compression bandaging. J Wound Care.

[13] Labropoulos N (2019). How does chronic venous disease progress from the first symptoms to the advanced stages? A review. Adv Ther.

[14] Chwała M, Szczeklik W, Szczeklik M, Aleksiejew-Kleszczyński T, Jagielska-Chwała M (2015). Varicose veins of lower extremities, hemodynamics and treatment methods. Adv Clin Exp Med.

[15] Lin F, Zhang S, Sun Y, Ren S, Liu P (2015). The management of varicose veins. Int Surg.

[16] Rodriguez Santos F, Loson V, Coria A (2020). Secondary ablation of recanalized saphenous vein after endovenous thermal ablation. Ann Vasc Surg.

[17] Campbell B (2006). Varicose veins and their management. BMJ.

[18] Sotiris D, Pallotta G, Nittari G, Amenta F (2020). An original approach for the treatment of varicose veins of the lower limbs. J Clin Aesthet Dermatol.

[19] Jones RH, Carek PJ (2008). Management of varicose veins. Am Fam Physician.

[20] Azar J, Rao A, Oropallo A (2022). Chronic venous insufficiency: a comprehensive review of management. J Wound Care.

[21] Subramonia S, Lees TA (2007). The treatment of varicose veins. Ann R Coll Surg Engl.

[22] Beale RJ, Gough MJ (2005). Treatment options for primary varicose veins-a review. Eur J Vasc Endovasc Surg.

[23] Broderick C, Watson L, Armon MP (2021). Thrombolytic strategies versus standard anticoagulation for acute deep vein thrombosis of the lower limb. Cochrane Database Syst Rev.

[24] de Ávila Oliveira R, Riera R, Vasconcelos V, Baptista-Silva JC (2021). Injection sclerotherapy for varicose veins. Cochrane Database Syst Rev.

[25] Belramman A, Bootun R, Lane TR, Davies AH (2019). Endovenous management of varicose veins. Angiology.

[26] Paravastu SC, Horne M, Dodd PD (2016). Endovenous ablation therapy (laser or radiofrequency) or foam sclerotherapy versus conventional surgical repair for short saphenous varicose veins. Cochrane Database Syst Rev.

[27] Rigby KA, Palfreyman SJ, Beverley C, Michaels JA (2002). Surgery for varicose veins: use of tourniquet. Cochrane Database Syst Rev.

[28] Andrews RH, Dixon RG (2021). Ambulatory phlebectomy and sclerotherapy as tools for the treatment of varicose veins and telangiectasias. Semin Intervent Radiol.

[29] Nesbitt C, Bedenis R, Bhattacharya V, Stansby G (2014). Endovenous ablation (radiofrequency and laser) and foam sclerotherapy versus open surgery for great saphenous vein varices. Cochrane Database Syst Rev.

[30] Galanopoulos G, Lambidis C (2012). Minimally invasive treatment of varicose veins: endovenous laser ablation (EVLA). Int J Surg.

[31] Hartmann K (2020). Endovenous (minimally invasive) procedures for treatment of varicose veins: the gentle and effective alternative to high ligation and stripping operations. Hautarzt.

[32] Rudarakanchana N, Berland TL, Chasin C, Sadek M, Kabnick LS (2012). Arteriovenous fistula after endovenous ablation for varicose veins. J Vasc Surg.

[33] Böhler K (2023). Minimally invasive catheters in varicose vein treatment: New gold standard? (Article in German). Dermatologie (Heidelb).

[34] Bootun R, Davies AH (2016). Long-term follow-up for different varicose vein therapies: is surgery still the best?. Phlebology.

[35] Whiteley MS (2022). Current best practice in the management of varicose veins. Clin Cosmet Investig Dermatol.

[36] Chiesa R, Marone EM, Limoni C, Volontè M, Petrini O (2007). Chronic venous disorders: correlation between visible signs, symptoms, and presence of functional disease. J Vasc Surg.

[37] Epstein D, Onida S, Bootun R, Ortega-Ortega M, Davies AH (2018). Cost-effectiveness of current and emerging treatments of varicose veins. Value Health.

[38] Nitecki S, Kantarovsky A, Portnoy I, Bass A (2006). The contemporary treatment of varicose veins (strangle, strip, grill or poison). Isr Med Assoc J.

[39] Bellmunt-Montoya S, Escribano JM, Pantoja Bustillos PE, Tello-Díaz C, Martinez-Zapata MJ (2021). CHIVA method for the treatment of chronic venous insufficiency. Cochrane Database Syst Rev.

[40] Zhao C, Li D, Xie C (2023). Efficacy and safety of ultrasound-guided foam sclerotherapy combined with endoluminal radiofrequency closure in patients with varicose veins of lower extremities. Int Wound J.

